# Population structure, genetic diversity and genomic selection signatures among a Brazilian common bean germplasm

**DOI:** 10.1038/s41598-021-82437-4

**Published:** 2021-02-03

**Authors:** Jessica Delfini, Vânia Moda-Cirino, José dos Santos Neto, Paulo Maurício Ruas, Gustavo César Sant’Ana, Paul Gepts, Leandro Simões Azeredo Gonçalves

**Affiliations:** 1 Agronomy Department, Universidade Estadual de Londrina (UEL), Londrina, 86051-900 Brazil; 2 Plant Breeding, Instituto de Desenvolvimento Rural do Paraná-Iapar-Emater (IDR-Paraná), Londrina, 86047-902 Brazil; 3 Biology Department, Universidade Estadual de Londrina (UEL), Londrina, 86051-900 Brazil; 4 Tropical Melhoramento and Genética (TMG), Londrina, 86188-000 Brazil; 5 Section of Crop and Ecosystem Sciences, Department of Plant Sciences, University of California, Davis, 95616-8780 USA

**Keywords:** Plant biotechnology, Agricultural genetics, Agricultural genetics, Genetic markers, Plant breeding

## Abstract

Brazil is the world's largest producer of common bean. Knowledge of the genetic diversity and relatedness of accessions adapted to Brazilian conditions is of great importance for the conservation of germplasm and for directing breeding programs aimed at the development of new cultivars. In this context, the objective of this study was to analyze the genetic diversity, population structure, and linkage disequilibrium (LD) of a diversity panel consisting of 219 common bean accessions, most of which belonging to the Mesoamerican gene pool. Genotyping by sequencing (GBS) of these accessions allowed the identification of 49,817 SNPs with minor allele frequency > 0.05. Of these, 17,149 and 12,876 were exclusive to the Mesoamerican and Andean pools, respectively, and 11,805 SNPs could differentiate the two gene pools. Further the separation according to the gene pool, bayesian analysis of the population structure showed a subdivision of the Mesoamerican accessions based on the origin and color of the seed tegument. LD analysis revealed the occurrence of long linkage blocks and low LD decay with physical distance between SNPs (LD half decay in 249 kb, corrected for population structure and relatedness). The GBS technique could effectively characterize the Brazilian common bean germplasms, and the diversity panel used in this study may be of great use in future genome-wide association studies.

## Introduction

The common bean (*Phaseolus vulgaris* L.) is one of the five cultivated species of the *Phaseolus* genus and is one of the most consumed legumes worldwide. It is the most important legume grain for direct human consumption and the main source of protein and micronutrients in several countries^1^. Globally, around 31 million tons of bean grains are produced per year, with the Americas accounting for 32.4% of the total production. Brazil is the world's largest producer of common bean, and other countries that are among the largest producers are India, Myanmar, China, United States, and Mexico^2^.

The common bean is known to have originated in Mexico and the Southern Andes, where it was domesticated independently to give rise to two gene pools, i.e., the Andean and Mesoamerican groups, which are morphologically and genetically different^3–6^. Different parts of the world prefer either the Andean or Mesoamerican grains. The Mesoamerican common beans are more common in North America, Central America, and the lowland part of South America, whereas the Andean common beans are preferred in parts of Africa, Europe, and Andean part of South America^7,8^. In Brazil, Mesoamerican common beans are preferred, of which the carioca and black beans represent the most consumed commercial groups^9,10^. Carioca beans are the most widely produced in Brazil, accounting for approximately 70% of the national common bean production, whereas black beans represent about 15% of the total production^11^.

Genetic diversity studies are of great importance for breeding programs, as they provide valuable information for effective conservation and application of available germplasm^12^. Such studies facilitate the understanding of genetic relationships between accessions, identification of redundancies and admixtures in the germplasm, and determination of genitor pairs with adequate genetic distance.

Molecular markers have been widely used in plant breeding programs. Several different types of markers are available; however, their applications have been restricted in the past due to limitations such as low density, labor intensity, technical requirements, and high cost of large-scale analysis^13,14^. The advent of next-generation sequencing (NGS) technologies has resulted in an exponential increase in the number of genetic variants that can be discovered in a single experiment^15^. The publication of the complete genome sequence of the common bean by Schmutz et al.^5^ facilitated the discovery of single nucleotide polymorphisms (SNPs) and genetic mapping, further allowing the construction of maps from short reads of different genotypes using the genome sequence as a reference^16^.

Among the NGS methods, the genotyping by sequencing (GBS) technique has emerged as a new approach to mitigate the constraints of previously employed markers^17^. GBS is a robust, high-performance, cost-effective, and simple technique for obtaining thousands of markers from a large number of individuals, and allows the identification of SNPs using a reduced representation library^18–20^. SNPs are the most abundant and universal sequence variations in all genomes, which makes them very useful markers for genetic analyses in plants^21^.

The GBS technique is often employed in plant breeding, and is frequently used in genetic diversity studies, mapping (linkage and association) studies, and genomic selection (GS)^13^. Genome-wide association studies (GWAS) are a powerful tool for identifying candidate genomic regions associated with traits of interest. Some of the most important parameters for successful GWAS are the representativity of the diversity panel, the size of the panel, the levels and genomic distribution of linkage disequilibrium (LD), and the population structure or genetic relationships among individuals^22–24^. The diversity panel should represent most of the available genetic and phenotypic diversity, and LD should be analyzed to determine the density of markers required for GWAS^25^.

Studies on genetic diversity and population structure have already been conducted for several crops, including wheat^26–28^, flaxseed^29^, pepper^30^ and rice^31^. Several diversity panels have also been developed for the common bean crop, including accessions from different regions of the world^32–37^. Based on these initial studies, several GWAS have further been conducted for different traits of interest, such as yield, plant architecture, nutritional content of grains, cooking time, resistance to diseases, and tolerance to abiotic factors^7,32,37–47^. Some GWAS have been conducted in Brazil^37,40,48^, however, panels consisting of different genotypes, can contribute to a better understanding about the genetic diversity and relationships of the germplasm available for genetic breeding.

In view of the above, the objective of the present study was to analyze the genetic diversity, population structure, and LD of the Brazilian Diversity Panel (BDP), which is a common bean diversity panel representing a large proportion of the genetic diversity of Brazilian common bean populations. It is composed mainly of materials from the carioca and black bean commercial groups, which are the most consumed cultivars in the country, and is expected to be used for GWAS in the future.

## Results

### Genotyping by sequencing

Using the GBS method optimized for common beans by Ariani et al.^18^, a total of 392,585,199 good barcoded reads were obtained from the sequenced accessions, of which 364,454,550 could be aligned with the Andean reference genome (G19833^5^), resulting in an average mapping rate of 93%. Initially, 461,199 SNPs were obtained, of which 49,817 SNPs were retained after filtering. Eleven accessions had a low rate of genotyping (less than 10% of genotyped positions) and were excluded from the BDP, for this reason 219 accessions were used in the subsequent analyses.

**Figure 1:**
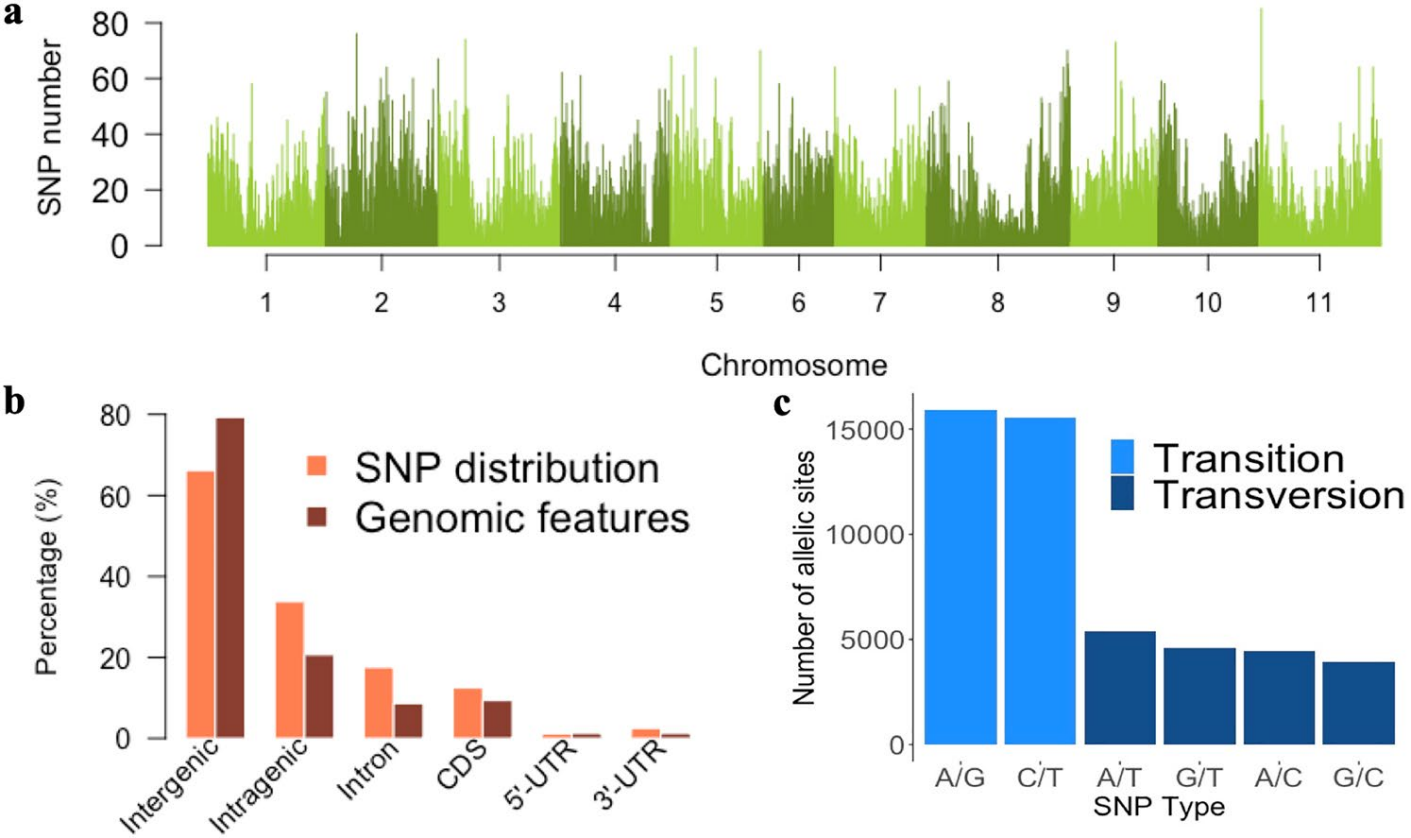
Identification and annotation of 49,817 single nucleotide polymorphisms (SNPs) obtained from the genotyping of 219 common bean accessions. (**a**) Distribution of SNP density along the common bean genome in a 200 kb sliding window. (**b**) Annotation of SNPs and proportion of genomic traits. (**c**) Transversion/transition ratio. Figure produced in R v.4.0.

SNPs were unevenly distributed throughout the genome, and fewer SNPs were observed in regions near the centromere than in regions near the telomeres on the chromosome (Fig. 1a). The mean number of SNPs per chromosome was 4528, ranging from 3361 to 5910 SNPs on the Pv06 and Pv02 chromosomes, respectively (Table 1). Physical chromosome length was positively correlated with the number of SNPs (r = 0.74, p < 0.01).

**Table 1:** Number of SNPs in each of the 11 common bean chromosomes in the set of 219 accessions from the Brazilian Diversity Panel.

Chromosome	Physical length (Mb)^a^	Number of genes^a^	Total number of SNPs	Tagged genes
Pv01	52.20	2779	4718	787
Pv02	49.04	3435	5910	1111
Pv03	52.28	3058	4983	874
Pv04	45.96	1890	4650	596
Pv05	40.82	1928	4321	529
Pv06	31.97	2295	3361	721
Pv07	51.75	2895	3903	853
Pv08	59.66	3023	5635	914
Pv09	37.47	2719	3735	716
Pv10	43.27	1721	3939	512
Pv11	50.37	2253	4662	653
Total	–	27,996	49,817	8266

^a^Information obtained from the *EnsemblPlants* website (https://plants.ensembl.org).

Of the total SNPs obtained, 33.8% were located in intragenic regions (17.5% in intron and 16.3% in exons), 12.5% in coding DNA sequences, 1.2% in 5′ UTR regions, and 2.6% in 3′ UTR regions (Fig. 1b). Thirty percent of the annotated genes in the reference genome of *Phaseolus vulgaris* v2.0 were tagged by at least one SNP (tagged genes) (Table 1). A positive correlation between the number of genes and tagged genes per chromosome was observed (r = 0.96, p < 0.01).

Of the different types of polymorphism, transitions (63.1%) were more frequent than transversions (36.9%), resulting in a transition/transversion rate of 1.71 (Fig. 1c). The percentages of A/G and C/T transitions were very similar (32% and 31%, respectively), as were those of polymorphism due to A/T, A/C, G/T, and G/C transversions (11%, 9%, 9%, and 8%, respectively). Considering only the SNPs inside genes the transition/transversion rate was 1.27, smaller compared to the overall rate, for the reason that the percentage of transversions (44.1%) was greater than the overall.

### Genetic diversity and population structure

**Figure 2:**
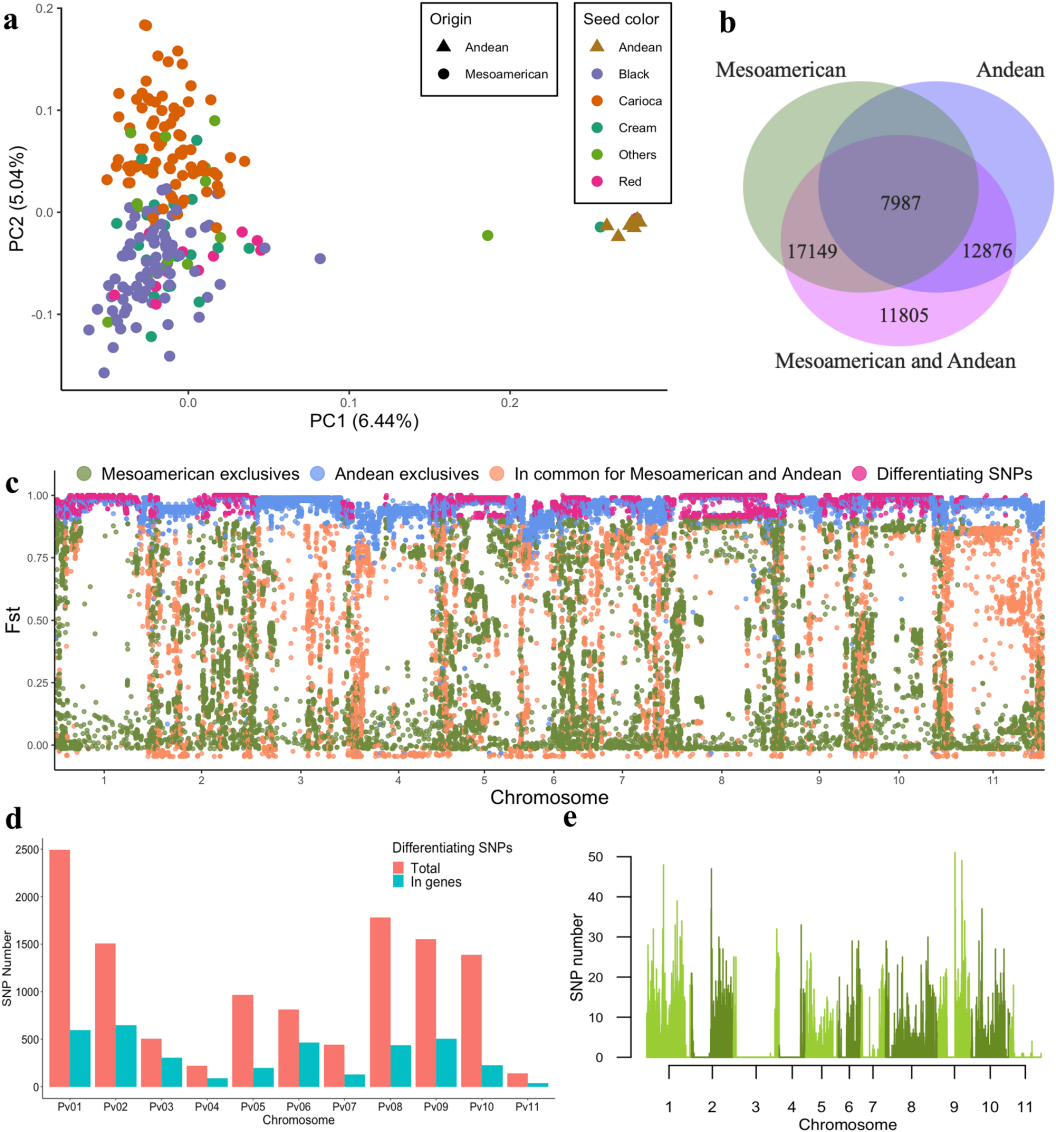
Genetic differentiation between Andean and Mesoamerican gene pools. (**a**) Principal component analysis of 219 accessions of Andean and Mesoamerican origin including different commercial groups (black, carioca, cream, red, etc.). (**b**) Venn diagram of the total set of SNPs and SNPs belonging to the Andean and Mesoamerican groups. (**c**) Distribution of the *Fst* values of each SNP (colored according to the population in which they occur). (**d**) Total number of differentiating SNPs on each chromosome and number of differentiating SNPs located within genes. (**e**) Distribution of the 11,805 differentiating SNPs of the Andean and Mesoamerican groups along the common bean genome in a 200 kb sliding window. Figures (**a**), (**c**), (**d**) and (**e**) produced in R v.4.0 and Figure b produced in JVENN (http://jvenn.toulouse.inra.fr).

The population structure of all accessions included in the BDP were analyzed using 819 SNPs that were retained after LD filtering (*r*^2^ < 0.2). The results of the principal component analysis (PCA) showed that the accessions could be segregated into two distinct groups, based on the gene pools (Andean and Mesoamerican) (Fig. 2a).

**Figure 3:**
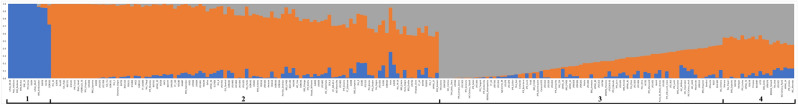
Analysis of the population structure using 219 accessions belonging to the Brazilian common bean diversity panel with K = 3: (1) corresponds to the group of common beans of Andean origin; (2) mostly formed by Mesoamerican accessions of black, cream, red, and other seed tegument colors; (3) mostly formed by Mesoamerican accessions from the carioca commercial group; and (4) mostly formed by Mesoamerican accessions with membership coefficient < 0.6 for the previous groups. Figure produced in R v.4.0.

The two gene pools were also segregated in the Bayesian population structure analysis. However, based on the ΔK^49^ criterion, the number of groups (K) with the highest value of ΔK was three (K = 3), which demonstrated a subdivision of the Mesoamerican group (Fig. 3). Based on the membership coefficient (≥ 0.6), 90.9% of the accessions could be assigned to a specific group, and only 20 accessions were categorized as admixtures. The accessions of Andean origin formed a group, and the Mesoamerican accessions were divided into two distinct groups and the admixture group. In the two Mesoamerican groups formed solely by individuals with a membership coefficient ≥ 0.6, the accessions were distinguished by the color of the seed tegument; one group was composed primarily of carioca-type grain accessions, whereas the other group included accessions with black, purple, red, cream, and other tegument colors. The admixture group comprised accessions that had resulted from hybridization between the previous two groups. The accessions of commercial groups other than black and carioca (i.e., purple, red, cream, and others) were predominantly grouped with the black commercial group; however, there was a tendency to cluster according to the color of the flower, which is purple in the black group, white in the carioca group, and variable (white, pink, and purple) in other accessions. Three accessions initially identified as Mesoamerican were assigned to the Andean group in these analyses and were therefore treated as Andean in subsequent analyses.

Removal of accessions of Andean origin from the panel left 207 accessions of the Mesoamerican origin. Among these 207 accessions, 25,136 SNPs with MAF > 0.05 could be identified, i.e., the number of SNPs per chromosome was reduced on average by 50% relative to the number of SNPs identified when the Andean accessions were included in the panel. The chromosomes exhibiting the greatest reduction in the number of SNPs were Pv05 and Pv11, whereas Pv09 and Pv01 presented the smallest reduction.

### Genetic differentiation between Andean and Mesoamerican gene pools

The two gene pools shared 7987 SNPs, whereas 17,149 and 12,876 SNPs were unique to the Mesoamerican and Andean groups (Fig. 2b), respectively. The mean pairwise fixation index (*Fst*) for each of these SNP groups was 0.39, 0.34, and 0.94, respectively (Fig. 2c). A total of 11,805 SNPs differentiating the Andean and Mesoamerican groups were detected, with a mean *Fst* of 0.97. The mean *Fst* between the Andean and Mesoamerican pools was 0.77 when all the SNPs were included. The Mesoamerican group showed greater mean nucleotide diversity (π = 0.31) than the Andean group (π = 0.22). Regarding Tajima’s D, the Mesoamerican gene pool showed a positive value (D = 1.50), while the Andean gene pool showed a negative value (D = − 0.50) (Table 2).

**Table 2:** Nucleotide diversity (π), Tajima’s D and weighted *Fst* estimated in the Brazilian common bean diversity panel in relation to different centers of origin, seed colors, and institutions of origin.

	N	SNPs	π	D	*Fst*^1^		
**Origin**					Andean		
Mesoamerican	207	25,136	0.31	1.50	0.77		
Andean	12	20,863	0.22	− 0.50			
**Seed tegument color**					Black	Red	Cream
Cream with brown stripes (Carioca)	85	22,275	0.32	1.32	0.12	0.22	0.12
Black	78	22,289	0.31	1.23		0.10	0.03
Red	11	18,447	0.35	0.69			0.08
Cream	19	27,579	0.29	0.40			
**Institutions of origin**^2^					EMBRAPA	IAC	IAPAR
CIAT	45	21,676	0.34	1.28	0.06	0.12	0.09
EMBRAPA	29	22,183	0.34	1.08		0.09	0.01
IAC	14	28,577	0.30	0.44			0.06
IAPAR	84	22,735	0.32	1.35			

*N *number of accessions, *SNPs *number of SNPs, *π *nucleotide diversity, *D *Tajima’s D statistics. ^1^Weir and Cockerham, 1984. ^2^*CIAT *International Center for Tropical Agriculture (Centro Internacional de Agricultura Tropical), *EMBRAPA *Brazilian Agricultural Research Corporation (Empresa Brasileira de Pesquisa Agropecuária), *IAC *Agronomic Institute of Campinas (Instituto Agronômico de Campinas), *IAPAR *Rural Development Institute of Paraná–IAPAR–EMATER (Instituto de Desenvolvimento Rural do Paraná).

Most SNPs that differentiate the Andean and Mesoamerican pools were located on chromosomes Pv01 (2492 SNPs), Pv08 (1781 SNPs), Pv09 (1554 SNPs), Pv02 (1506 SNPs), and Pv10 (1387 SNPs) (Fig. 2d,e), and 30.8% were located within genes, with 2187 genes including at least one differentiating SNP. Most of these SNPs inside genes were located on chromosomes Pv02 (648 SNPs), Pv01 (595 SNPs), Pv09 (504 SNPs), Pv06 (476 SNPs), and Pv08 (435 SNPs) chromosomes (Fig. 2d). Among the SNPs located in coding regions, 26% were synonymous SNPs and 74% were non-synonymous (being 68% missense variants). Of the genes containing the differentiating SNPs, 279 were putative candidates for domestication, of which 179 are known to be involved in the domestication of the Mesoamerican group, 91 in that of the Andean group, and 9 in the domestication of both these groups^5^.

### Genetic differentiation among the Mesoamerican accessions

**Figure 4:**
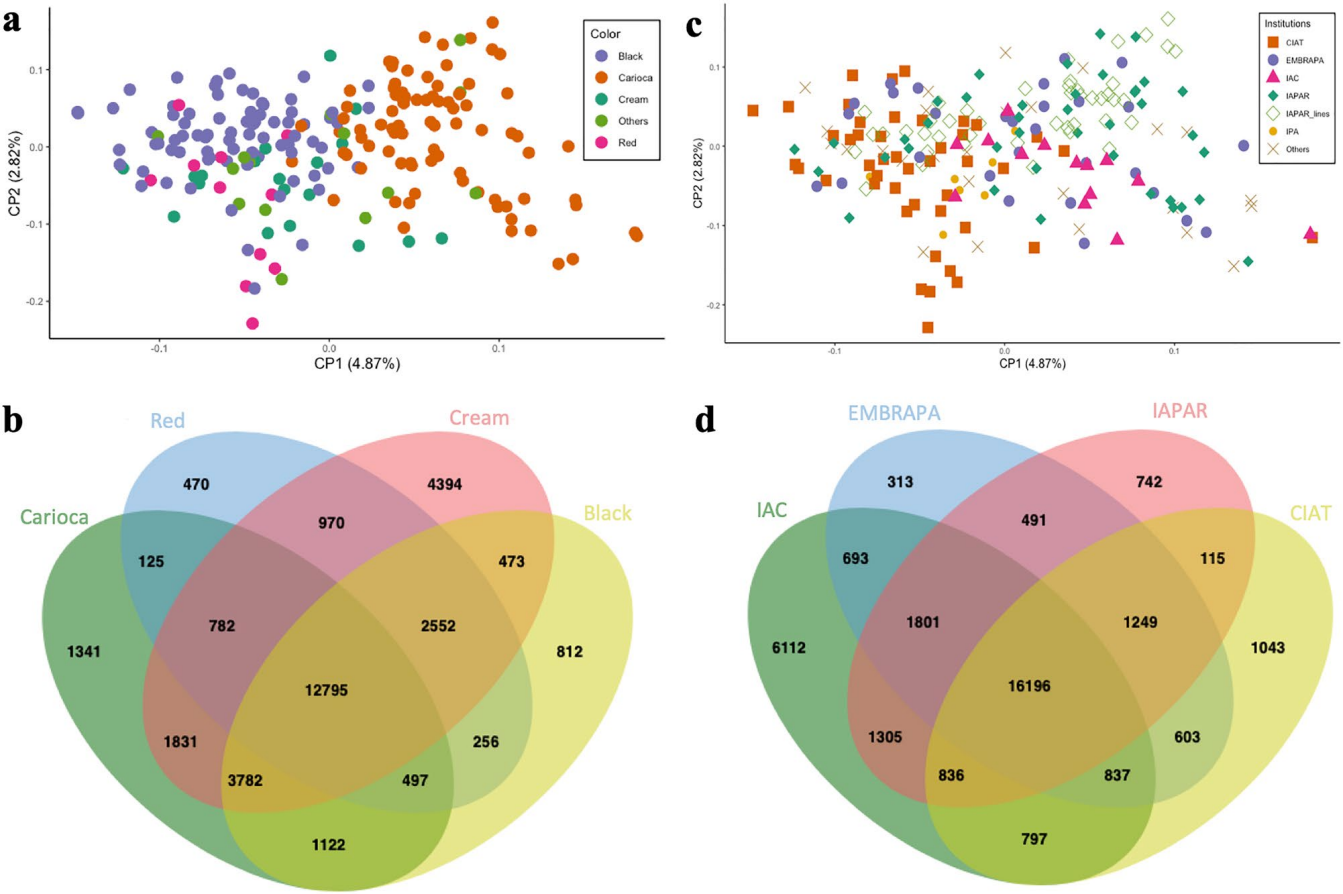
Principal component analyses and Venn diagrams. (**a**) Principal component analysis of 207 accessions of common beans of Mesoamerican origin with different seed tegument colors. (**b**) Venn diagram for the different sets of SNPs related to seed tegument color. (**c**) Principal component analysis of 207 accessions of common beans from different research institutions. (**d**) Venn diagram for the different sets of SNPs related to the institutions of origin. Figure (**a**) and (**c**) produced in R v.4.0 and Figures b and d produced in JVENN (http://jvenn.toulouse.inra.fr).

**Figure 5:**
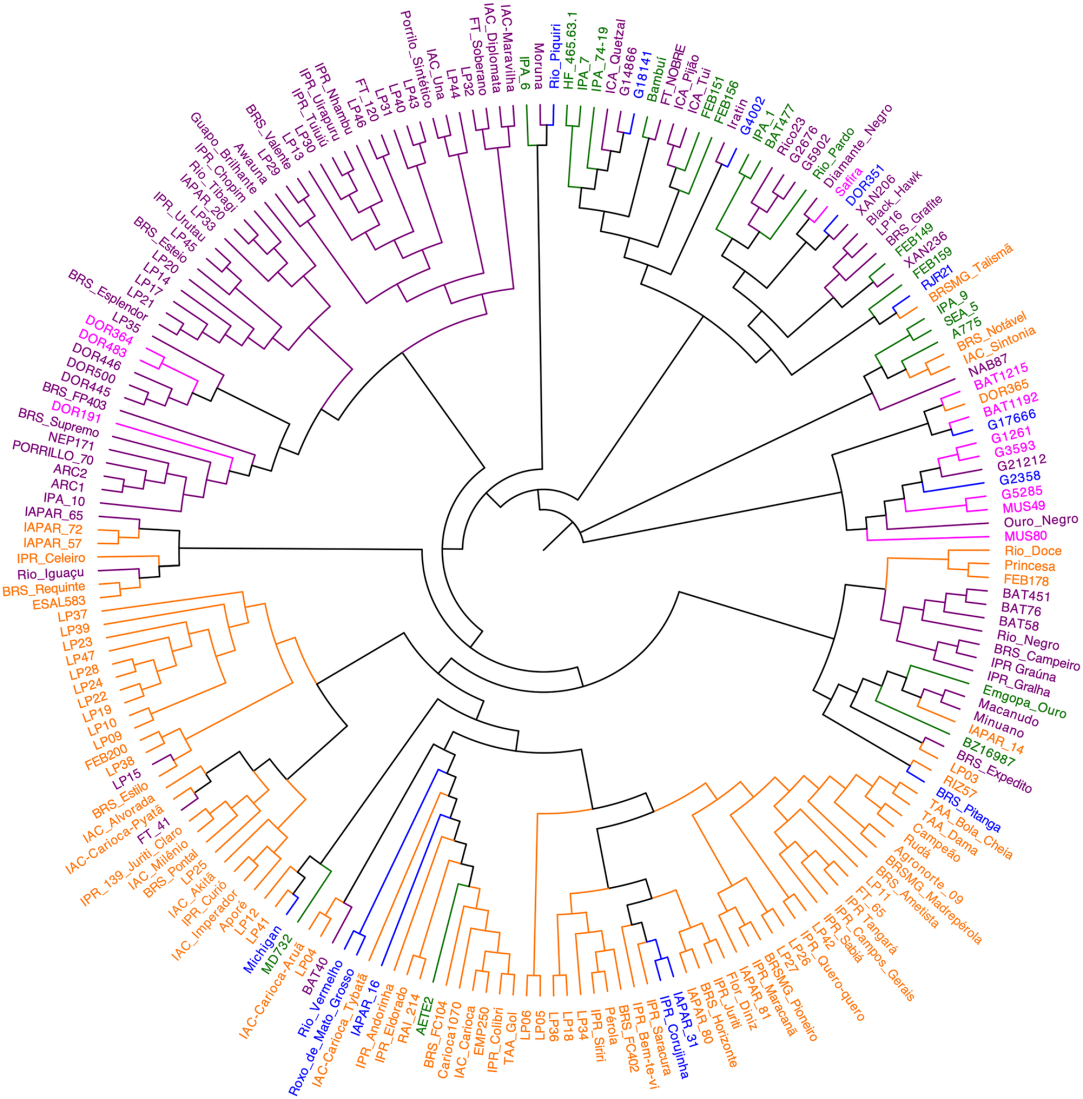
Dendrogram showing the genetic relatedness among 207 common bean accessions belonging to the Brazilian Diversity Panel. The different colors identify the accessions according to the color of the seed tegument. Purple = black tegument, Orange = carioca-type tegument, Green = cream tegument, Pink = red tegument, and Blue = others. Figure produced in FigTree v1.4.4.

As seen in the PCA and Bayesian analysis of population structure, the accessions of Mesoamerican origin were also segregated into two main groups in the phylogenetic tree, based on the tegument color, with one group consisting of the carioca commercial group and the other including the accessions with black, cream, red, white and purple tegument (Figs. 4a, 5).

The separation of Mesoamerican individuals by seed color showed that each group had a variable number of SNPs, and only 12,795 SNPs were common to all these color groups (Fig. 4b). The cream-colored accessions exhibited the highest number of SNPs (27,579) and the lowest π (0.29) value. The red-colored group had the highest π (0.35), whereas π values of the carioca and black groups were similar (0.32 and 0.31, respectively) (Table 2). According to *Fst,* the carioca and red groups were the most different, with an *Fst* value of 0.22, whereas comparisons between the other colors yielded low *Fst* values. The Tajima’s D values were all positive in relation to the seed tegument color as well as for the institution of origin (Table 2).

Regarding the institution of origin, a clustering trend was observed for the accessions of the International Center for Tropical Agriculture (CIAT), Agronomic Institute of Pernambuco (IPA), and the more recent inbred lines of the Rural Development Institute of Paraná—IAPAR–EMATER (IAPAR) (Fig. 4c). The number of SNPs was variable for each institution; however, the π was similar. A total of 16,196 SNPs was shared in the accessions of all institutions, whereas 8210 were exclusive, i.e., belonged to only one institution (Fig. 4d). The Agronomic Institute of Campinas (IAC) accessions presented the highest number of exclusive markers (6112), whereas the Brazilian Agricultural Research Corporation (EMBRAPA) accessions included only 313 unique markers. Comparison of the accessions from different institutions did not yield high differentiation indexes (*Fst*), with the highest value being observed between CIAT and IAC (0.12) and the lowest value between IAPAR and EMBRAPA (0.01) (Table 2).

### Linkage disequilibrium

**Figure 6:**
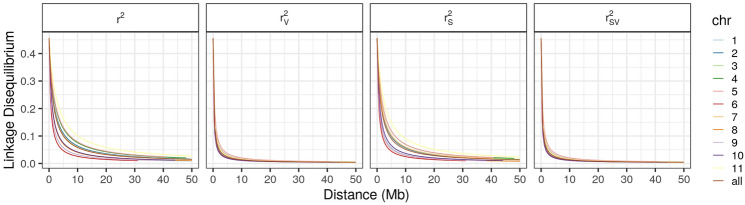
Analysis of linkage disequilibrium (LD) decay as a function of physical distance without correction (*r*^2^), and after correcting for population structure (*r*^2^_*s*_), relatedness (*r*^2^_*v*_), and for both population structure and relatedness (*r*^2^_*vs*_). Figure produced in R v.4.0.

LD decay and half-decay distances were calculated for individual chromosomes and for the whole genome. In both cases, the differences between conventional *r*^2^ and population structure-corrected *r*^2^ (*r*^2^_*s*_) were small. Considering the whole genome (all chromosomes), the half-decay distance was 1361 kb and 1180 kb for *r*^2^ and *r*^2^_*s*_, respectively. The *r*^2^ was remarkably different when compared with *r*^2^_*v*_ (*r*^2^ corrected for relatedness) and *r*^2^_*vs*_ (*r*^2^ corrected for population structure and relatedness) (Fig. 6). The latter two measures exhibited very similar decay values, with half-decay occurring at 249 kb.

In the analysis of *r*^2^_*v*_ and *r*^2^_*vs*_ of individual chromosomes, the half-decay distance ranged from 183 to 397. The highest decay values were noted for chromosomes Pv10, Pv08, Pv01, and Pv06 chromosomes (183, 187, 193 and 198 kb, respectively), whereas the lowest decay values were presented by chromosomes Pv05, Pv04, Pv07, and Pv09 (397, 322, 317 and 310, respectively) (Fig. 6).

## Discussion

The common bean is a very important crop in Brazil and is cultivated in all states of the country, mainly by family farmers. Considering the history of common bean cultivation in the country, domesticated common beans are highly diverse, although Brazil is not a primary center of diversity^50,51^. In this context, the present study was developed to understand the genetics and population structure of a newly created common bean diversity panel that includes a large part of the diversity of the most consumed common bean types in Brazil. These results will assist future GWAS for determining genomic regions or genes associated with several economically important traits.

The GBS methodology proposed by Ariani et al.^18^ was used in this study, which could effectively detect numerous SNPs in the analyzed accessions. These authors found that the *CviAII* enzyme was more effective than the commonly used *ApeKI* enzyme. As a methylation-insensitive enzyme, *CviAII* exhibited a higher number of restriction sites and acted preferentially on non-repetitive parts of the genome, allowing the identification of thousands of markers spaced unevenly throughout the common bean genome, with a density distribution resembling that of the distribution of genes.

Initially, 461,199 SNPs were identified. However, 89% of the markers did not satisfy the filtering criteria (Non-biallelic, indels, MAF < 0.05, coefficient of inbreeding < 0.9 and less than 10% of genotyped positions) and were not used in subsequent analyses. Polymorphisms were widely distributed across the 11 chromosomes and were highly correlated with the length and number of genes on each chromosome. The transition/transversion rate was consistent with that observed in other studies on common bean and other species^28,35,52,53^. Transitions are usually more frequent than transversions in several species, which indicates that the former are better tolerated during natural selection, which may be due to the fact that they are synonymous mutations in protein-coding sequences^29,54^.

Because LD may affect the inference of the population structure, an LD filter was further applied, which resulted in a decrease of the number of SNPs. This is due to the fact that the common bean is an autogamous plant with very long blocks of markers in LD^35,55,56^.

Genetic differentiation between common bean accessions based on the gene pool has been well documented in several previous studies^3,34,35,57–60^. The relationship between the genetic similarity of the Mesoamerican accessions and the color of the seed tegument was also observed by Valdisser et al.^61^ and Gioia et al.^62^. In Brazil, breeding programs for the carioca and black commercial groups have different objectives^63^. Moreover, genetic breeding of the carioca group is much more advanced than that of the black group, because of its greater importance in the country due to consumers and market preferences. Efforts to improve the carioca bean are directed towards the grain size traits, to satisfy the consumers’ preference for larger grains. However, the grain size is negatively correlated with yield, in case of the black group, selection is based mainly on yield, resulting in cultivars with smaller grains^64^.

Several SNPs exclusive to either of the gene pools were observed, in addition to the differentiating SNPs between the two pools. Other authors have also reported that the proportion of polymorphic loci tends to be higher in populations composed of accessions from the two centers of origin, and it tends to decrease when they are studied separately^61,65,66^. The two gene pools differ in both phenotypic and molecular characteristics, which is supported by the high rates of genetic differentiation obtained in the present analysis and in other studies^50,51,61,67^. In addition, the Mesoamerican gene pool exhibits higher nucleotide diversity than the Andean, possibly because a strong bottleneck occurred during the dispersal of Southern Andean common beans from Mesoamerica, which drastically reduced its nucleotide diversity^4,5,33,34,60,67^.

To identify genomic signatures of selection between the Andean and Mesoamerican pools, the *Fst* was estimated for each SNP. The *Fst* of nucleotide positions that were polymorphic only when the two gene pools were studied together was close to 1, and these SNPs were therefore highly discriminating between the gene pools. The *Fst* of SNPs present only in the Andean group was also high, similar to that of the discriminating markers. This may be due to the small number of Andean accessions included in this study.

There is significant evidence supporting the independent domestication of the Andean and Mesoamerican gene pools. Schmutz et al.^5^ identified 1835 candidate genes for domestication in the Mesoamerican group and 748 candidate genes in the Andean group. Of these, only 59 genes were common to both groups. These genes are mainly located on chromosomes Pv01, Pv02, Pv07, Pv09, and Pv10. In the present study, 11% of all the candidate genes for domestication harbored differentiating SNPs. These genes have also been identified in other studies aimed at finding selection signatures between the Andean and Mesoamerican^34,35^ accessions. The candidate genes for domestication are directly or indirectly associated with the main characteristics that distinguish the two gene pools, such as flowering time, plant size, and seed size.

The low rate of differentiation of the accessions based on the institution of origin may be related to the protocols of the breeding programs. Breeding programs tend to be conservative and almost always employ the Mesoamerican germplasm, with little exploration of exotic germplasms; in addition, they use a selected group of elite parents, which further narrows the genetic base^57,61–63^. Because of significant exchange of germplasm between institutions, there was no formation of well-defined groups among the accessions from the different institutions of origin^57^, only a trend for clustering was observed for accessions belonging to CIAT, IPA, and the more recent inbred lines of IAPAR.

LD measurement is very important in association mapping studies for identifying loci associated with quantitative traits. Importantly, the population structure and relatedness between the analyzed accessions may cause a bias in LD estimation. Frequent selection, admixture of populations, and crossing of a small number of cultivars in breeding programs reduces genetic diversity and affects LD patterns^68^. These factors can affect different genomic regions in several ways, which can introduce heterogeneity of LD through the genome. This makes the resolution and power achieved in GWAS dependent on the species and the population under study. LD decay is slower in autogamous species, such as common bean and soybean, in which recombination is less effective than in allogamous species^68,69^.

In this study, the LD corrected for population structure (*r*^2^_*s*_) was not significantly different from the conventional *r*^2^. However, *r*^2^_*v*_ and *r*^2^_*vs*_ exhibited a faster LD decay when compared with the conventional *r*^2^. The fact that *r*^2^_*v*_ was considerably lower than conventional *r*^2^ demonstrates the need to remove the effect of relatedness to reduce the overestimation of LD. The similarity between the estimated *r*^2^ and *r*^2^_*s*_ (LD half-decay with 296 kb) shows that the BDP is not highly structured, which is consistent with the results of other studies on common bean diversity panels^40,51,57^. As observed by Diniz et al.^57^ in panels composed mainly of improved genotypes, the degree of relatedness between individuals was very high.

The present study demonstrated that GBS is a powerful approach for analyzing the population structure and genetic diversity in common bean. The newly developed diversity panel, which represents a large proportion of the Brazilian common bean diversity, exhibited high genetic diversity, and was shown to be adequate for future studies to identify genomic regions related to traits of interest (GWAS).

## Methods

### Plant material

The BDP, including 230 common bean accessions that represent a large component of the common bean genetic diversity in Brazil, was used in this study (Table S1). The diversity panel is composed of modern and old cultivars developed between 1968 and 2019 by different research institutions (Table 2), in addition to inbred lines and landraces, all of which belong to the germplasm bank of the Rural Development Institute of Paraná –IAPAR–EMATER (IAPAR). Among the CIAT accessions present in this panel, most of them are inbred lines from breeding programs directed to the needs of Brazil and/or are accessions that compose the genealogy of cultivars developed by Brazilian institutions. Most accessions in this panel are of Mesoamerican origin, which exhibit significant diversity in the color of the seed tegument and include different commercial classes, with the carioca and black bean groups being the most representative. In addition, 10 accessions of Andean origin were included in this study for comparison.

### Genotyping by sequencing (GBS)

DNA extraction and the preparation of GBS libraries for sequencing was performed following the protocol developed for common bean by Ariani et al.^18^. DNA was extracted from lyophilized leaves collected from a single plant of each accession grown in a green house. The extracted DNA was purified using the Genomic DNA Clean and Concentrator kit (Zymo Research, CA, USA), according to the manufacturer's instructions. The DNA quality was checked using NanoDrop Lite (Thermo Fisher Scientific), and only samples with an absorbance ratio (A260/A280) greater than 1.7 were used for preparing the libraries. Genomic DNA was quantified using Quant-iT PicoGreen dsDNA Assay Kit (Thermo Fisher Scientific), and 100 ng of the DNA from each genotype was used for preparing the libraries.

The genomic DNA was digested using the restriction enzyme *CviAII* (recognition site C'ATG); after the preparation process, the samples were multiplexed into two libraries with up to 144 accessions each, including as control a blank sample and the genotype of *P. vulgaris* used to construct the reference genome (G19833) in each of the two libraries^67^. The presence of adapter dimers in the sequencing libraries was checked using DNA High Sensitivity Kit (Agilent 2100 Bionalyzer, Agilent Technologies).

The genomic libraries were sequenced using the Illumina HiSeq 4000 sequencer (Illumina, San Diego, CA, USA) with the 100-bp-single-end protocol, at the DNA Technologies and Expression Analysis Core Laboratory, located in the Genome Center, University of California, Davis, CA.

### Analysis of sequencing data

SNPs were called using the Tassel-5-GBS pipeline version 2^70^, with the standard software settings, except for the minimum quality score (-mnQs 20) and minimum count (-c 10) parameters. The obtained sequences were aligned with the reference genome of *Phaseolus vulgaris* v2.0 obtained from the Phytozome website (https://phytozome.jgi.doe.gov, accessed on March 10, 2019), using the Burrows-Wheeler Alignment (BWA) (-aln option) tool version 0.7.10^71^. Non-biallelic SNPs, and SNPs with indels, minor allele frequency (MAF) < 0.05, coefficient of inbreeding < 0.9, and those SNPs and accessions containing < 10% of genotyped positions were removed using VCFtools version 0.1.15^72^. Because common bean is an autogamous species, after the initial filtering, the occurrence of heterozygotes was insignificant, but heterozygous SNPs were treated as missing data, as they may indicate sequencing errors. After filtering, the SNPs were imputed using Beagle software version 5^73^, and only SNPs anchored to chromosomes in the common bean reference genome were used.

The SNPs were annotated according to the common bean genomic annotation (GFF3 file, version 2.1) available on the Phytozome website (https://phytozome.jgi.doe.gov, accessed on January 10, 2019), using a custom R^74^ script developed by Hu et al.^75^ (https://github.com/zhenbinHU/Sorghum_SNP_dataset, accessed on June 17, 2019).

### Genetic diversity and population structure

The 219 accessions belonging to the BDP that passed by the quality control mentioned above were included in the initial analyses. The population structure was inferred using the Bayesian clustering algorithm in Structure v2.3.4^76^ software from the command line python program StrAuto^77^. The admixture model with 50,000 burn-ins, 200,000 MCMC, and 10 replications for hypothetical numbers of subpopulations (K) between 1 and 10 was used. The statistical parameter ΔK^49^ was used to determine the number of groups. Only the accessions with a membership coefficient equal or higher than 0.6 were assigned to a genetic group, and those with membership coefficient lower than 0.6 were clustered in the admixture group. The admixture model assumes that the markers are not strongly linked; hence, the SNPs were filtered based on LD, using the indep-pairwise option of the PLINK^78^ software, and only SNPs with LD ≤ 0.2 were retained for population structure analysis. These data filtered for LD were also used for PCA, using the snpgdsPCA function of the SNPRelate^79^ package in R.

After verifying the center of origin, only individuals of Mesoamerican origin were retained, and the SNPs were again filtered to exclude monomorphics, SNPs with MAF < 0.05 and LD ≥ 0.2, using VCFtools version 0.1.15^72^ and PLINK^78^. These data were then used for PCA and population structure analysis, as previously described. In addition, phylogenetic inference was estimated using TASSEL v5^80^, based on identity-by-state (IBS) distance and using Neighbor-Joining as the clustering method. The generated tree was customized using FigTree v1.4.4^81^.

To detect molecular differences in relation to the center of origin, color of the seed tegument, and institution of origin, new files were created from the initial file (including all SNPs) containing the different groups, and only polymorphic SNPs and those with MAF > 0.05 were retained. Subsequently, a Venn diagram was constructed to detect the differentiating SNPs for each of the three parameters using the JVENN tool^82^. *Fst* index^83^, nucleotide diversity (π) and Tajima’s D^84^, were also calculated using VCFtools version 0.1.15^72^ and averaged on 100-kb genomic bins.

### Linkage disequilibrium

LD between SNPs was estimated using the LDcorSV^85^ package in R. This package corrects for the bias due to population structure and relatedness while estimating LD. In addition to the conventional *r*^2^, *r*^2^ corrected for population structure (*r*^2^_*s*_), *r*^2^ considering kinship (*r*^2^_*v*_), and *r*^2^ including both population structure and kinship (*r*^2^_*vs*_) were calculated. Only individuals belonging to the Mesoamerican group were used for these calculations. The STRUCTURE result at K = 2 for common beans of Mesoamerican origin was used as the population structure, and for relatedness the kinship matrix was calculated using the rrBLUP^86^ package in R. LD decay was calculated using the nonlinear method proposed by Hill and Weir^87^, and adjusted with the nls function in R.

## Supplementary Information

Supplementary Table S1.
