# Supplementary material for: Population structure, genetic diversity and genomic selection signatures among a Brazilian common bean germplasm
Source: Sci Rep. 2021 Feb 3;11:2964. doi: 10.1038/s41598-021-82437-4 (PMC7859210; doi:10.1038/s41598-021-82437-4)
Supplement: Supplementary file 1 — Supplementary Table S1. [file 41598_2021_82437_MOESM1_ESM.pdf]

Population structure, genetic diversity and genomic selection signatures among Brazilian common bean germplasms

Jessica Delfini[1,2], Vânia Moda-Cirino[2], José dos Santos Neto[1,2], Paulo Maurício Ruas[3], Gustavo César Sant'Ana[4], Paul Gepts[5] and Leandro Simões Azeredo Gonçalves[1*]

[1]Universidade Estadual de Londrina (UEL), Agronomy Department, Londrina, 86051-900, Brazil

[2] Instituto de Desenvolvimento Rural do Paraná – Iapar - Emater (IDR - Paraná), Plant Breeding, Londrina, 86047-902, Brazil

[3]Universidade Estadual de Londrina (UEL), Biology Department, Londrina, 86051-900, Brazil

[4]Tropical Melhoramento & Genética (TMG), Londrina, 86188-000, Brazil

[5]University of California, Department of Plant Sciences, Section of Crop and Ecosystem Sciences, 95616-8780, USA.

Supporting information

Additional supporting information may be found in the online version of this article.

Supplementary Table S1. List of accessions constituting the Brazilian Diversity Panel (BDP).

Supplementary Table S1. List of accessions constituting the Brazilian Diversity Panel (BDP).

Access name	Origin	Genetic Material	Developing institution[1]	Seed Color
BRS Ártico	Andean	Cultivar	EMBRAPA	White
BRS Embaixador	Andean	Cultivar	EMBRAPA	Red
BRS Radiante	Andean	Cultivar	EMBRAPA	Red (Others)
BRS Realce	Andean	Cultivar	EMBRAPA	Red (Others)
DRK 15	Andean	Cultivar	CIAT	Red
G6416	Andean	Breeding line	CIAT	Red
IPR Garça	Andean	Cultivar	IAPAR	White
KID44	Andean	Breeding line	CIAT	Red
LP01	Andean	Breeding line	IAPAR	Carioca
LP02	Andean	Breeding line	IAPAR	Carioca
Diamante Negro	Mesoamerican	Cultivar	EMBRAPA	Black
Aporé	Mesoamerican	Cultivar	EMBRAPA	Carioca
BRS Ametista	Mesoamerican	Cultivar	EMBRAPA	Carioca

BRS Campeiro	Mesoamerican	Cultivar	EMBRAPA	Black
BRS Esplendor	Mesoamerican	Cultivar	EMBRAPA	Black
BRS Esteio	Mesoamerican	Cultivar	EMBRAPA	Black
BRS Estilo	Mesoamerican	Cultivar	EMBRAPA	Carioca
BRS Expedito	Mesoamerican	Cultivar	EMBRAPA	Black
BRS FC104	Mesoamerican	Cultivar	EMBRAPA	Carioca
BRS FC402	Mesoamerican	Cultivar	EMBRAPA	Carioca
BRS FP403	Mesoamerican	Cultivar	EMBRAPA	Black
BRS Grafite	Mesoamerican	Cultivar	EMBRAPA	Black
BRS Horizonte	Mesoamerican	Cultivar	EMBRAPA	Carioca
BRS Notável	Mesoamerican	Cultivar	EMBRAPA	Carioca
BRS Pitanga	Mesoamerican	Cultivar	EMBRAPA	Purple
BRS Pontal	Mesoamerican	Cultivar	EMBRAPA	Carioca
BRS Requinte	Mesoamerican	Cultivar	EMBRAPA	Carioca
BRS Supremo	Mesoamerican	Cultivar	EMBRAPA	Black
BRS Valente	Mesoamerican	Cultivar	EMBRAPA	Black
BRS MGMadrepérola	Mesoamerican	Cultivar	EMBRAPA	Carioca
BRS MGPioneiro	Mesoamerican	Cultivar	EMBRAPA	Carioca
BRS MGTalismã	Mesoamerican	Cultivar	EMBRAPA	Carioca
Bambuí	Mesoamerican	Cultivar	EMBRAPA	Cream
Guapo Brilhante	Mesoamerican	Cultivar	EMBRAPA	Black
Macanudo	Mesoamerican	Cultivar	EMBRAPA	Black
Minuano	Mesoamerican	Cultivar	EMBRAPA	Black
Ouro Negro	Mesoamerican	Cultivar	UFV/EPAMIG	Black
Pérola	Mesoamerican	Cultivar	EMBRAPA	Carioca
Rudá	Mesoamerican	Cultivar	EMBRAPA	Carioca
Safira	Mesoamerican	Cultivar	EMBRAPA	Red
FT120	Mesoamerican	Cultivar	FT Sementes	Black
FT NOBRE	Mesoamerican	Cultivar	FT Sementes	Black
FT 41	Mesoamerican	Cultivar	FT Sementes	Black
FT 65	Mesoamerican	Cultivar	FT Sementes	Carioca
FT Soberano	Mesoamerican	Cultivar	FT Sementes	Black
IAC Akitã	Mesoamerican	Cultivar	IAC	Carioca
IAC Alvorada	Mesoamerican	Cultivar	IAC	Carioca
IAC Carioca	Mesoamerican	Cultivar	IAC	Carioca
IAC Diplomata	Mesoamerican	Cultivar	IAC	Black
IAC Formoso	Mesoamerican	Cultivar	IAC	Carioca
IAC Imperador	Mesoamerican	Cultivar	IAC	Carioca
IAC Milênio	Mesoamerican	Cultivar	IAC	Carioca

IAC Sintonia	Mesoamerican	Cultivar	IAC	Carioca
IAC Una	Mesoamerican	Cultivar	IAC	Black
IAC CariocaAruã	Mesoamerican	Cultivar	IAC	Carioca
IAC CariocaPyatã	Mesoamerican	Cultivar	IAC	Carioca
IAC CariocaTybatã	Mesoamerican	Cultivar	IAC	Carioca
IAC Maravilha	Mesoamerican	Cultivar	IAC	Black
Moruna	Mesoamerican	Cultivar	IAC	Black
IAPAR 16	Mesoamerican	Cultivar	IAPAR	Carioca (Others)
RAI214	Mesoamerican	Cultivar	IAPAR	Carioca
IAPAR 57	Mesoamerican	Cultivar	IAPAR	Carioca
IAPAR 65	Mesoamerican	Cultivar	IAPAR	Black
IAPAR 14	Mesoamerican	Cultivar	IAPAR	Carioca
IAPAR 20	Mesoamerican	Cultivar	IAPAR	Black
IAPAR 31	Mesoamerican	Cultivar	IAPAR	Carioca (Others)
IAPAR 72	Mesoamerican	Cultivar	IAPAR	Carioca
IAPAR 80	Mesoamerican	Cultivar	IAPAR	Carioca
IAPAR 81	Mesoamerican	Cultivar	IAPAR	Carioca
IPR 139 - JuritiClaro	Mesoamerican	Cultivar	IAPAR	Carioca
IPR Andorinha	Mesoamerican	Cultivar	IAPAR	Carioca
IPR Bem-te-vi	Mesoamerican	Cultivar	IAPAR	Carioca
IPR CamposGerais	Mesoamerican	Cultivar	IAPAR	Carioca
IPR Celeiro	Mesoamerican	Cultivar	IAPAR	Carioca
IPR Chopim	Mesoamerican	Cultivar	IAPAR	Black
IPR Colibri	Mesoamerican	Cultivar	IAPAR	Carioca
IPR Corujinha	Mesoamerican	Cultivar	IAPAR	Carioca (Others)
IPR Curió	Mesoamerican	Cultivar	IAPAR	Carioca
IPR Eldorado	Mesoamerican	Cultivar	IAPAR	Carioca
IPR Gralha	Mesoamerican	Cultivar	IAPAR	Black
IPR Graúna	Mesoamerican	Cultivar	IAPAR	Black
IPR Juriti	Mesoamerican	Cultivar	IAPAR	Carioca
IPR Maracanã	Mesoamerican	Cultivar	IAPAR	Carioca
IPR Nhambu	Mesoamerican	Cultivar	IAPAR	Black
IPR Quero-quero	Mesoamerican	Cultivar	IAPAR	Carioca
IPR Sabiá	Mesoamerican	Cultivar	IAPAR	Carioca
IPR Saracura	Mesoamerican	Cultivar	IAPAR	Carioca
IPR Siriri	Mesoamerican	Cultivar	IAPAR	Carioca
IPR Tangará	Mesoamerican	Cultivar	IAPAR	Carioca
FEB200	Mesoamerican	Breeding line	CIAT	Carioca
IPR Tuiuiú	Mesoamerican	Cultivar	IAPAR	Black

IPR Uirapuru	Mesoamerican	Cultivar	IAPAR	Black
IPR Urutau	Mesoamerican	Cultivar	IAPAR	Black
Rio Iguaçu	Mesoamerican	Cultivar	IAPAR	Black
Rio Negro	Mesoamerican	Cultivar	IAPAR	Black
Rio Pardo	Mesoamerican	Cultivar	IAPAR	Cream
Rio Piquiri	Mesoamerican	Cultivar	IAPAR	Brown
Rio Tibagi	Mesoamerican	Cultivar	IAPAR	Black
Rio Vermelho	Mesoamerican	Cultivar	IAPAR	Purple
Rio Doce	Mesoamerican	Cultivar	IAPAR	Carioca
Gordo	Mesoamerican	Cultivar	IPA	Cream
HF465.63.1	Mesoamerican	Cultivar	IPA	Cream
IPA1	Mesoamerican	Cultivar	IPA	Cream
IPA10	Mesoamerican	Cultivar	IPA	Black
IPA6	Mesoamerican	Cultivar	IPA	Cream
IPA7	Mesoamerican	Cultivar	IPA	Cream
IPA74-19	Mesoamerican	Cultivar	IPA	Cream
IPA9	Mesoamerican	Cultivar	IPA	Cream
Princesa	Mesoamerican	Cultivar	IPA	Carioca
TAA Bola Cheia	Mesoamerican	Cultivar	TAA	Carioca
TAA Dama	Mesoamerican	Cultivar	TAA	Carioca
TAA Gol	Mesoamerican	Cultivar	TAA	Carioca
Awauna	Mesoamerican	Cultivar	UEM	Black
Flor Diniz	Mesoamerican	Cultivar	UEM	Carioca
Rico23	Mesoamerican	Cultivar	UFV	Black
Campeão	Mesoamerican	Cultivar	Agristar	Carioca
Agronorte09	Mesoamerican	Cultivar	Agronorte	Carioca
ICA Pijão	Mesoamerican	Cultivar	ICA	Black
ICA Quetzal	Mesoamerican	Cultivar	ICA	Black
ICA Tui	Mesoamerican	Cultivar	ICA	Black
Iratin	Mesoamerican	Landrace	-	Black
Emgopa Ouro	Mesoamerican	Cultivar	Incaper	Cream
A775	Mesoamerican	Breeding line	CIAT	Cream
A779	Mesoamerican	Breeding line	CIAT	Cream
AETE2	Mesoamerican	Cultivar	IAC	Cream
ARC1	Mesoamerican	Breeding line	CIAT	Black
ARC2	Mesoamerican	Breeding line	CIAT	Black
BAT1215	Mesoamerican	Breeding line	CIAT	Red
BAT40	Mesoamerican	Breeding line	CIAT	Black
BAT41	Mesoamerican	Breeding line	CIAT	Black

BAT451	Mesoamerican	Breeding line	CIAT	Black
BAT58	Mesoamerican	Breeding line	CIAT	Black
BAT76	Mesoamerican	Breeding line	CIAT	Black
BAT1192	Mesoamerican	Breeding line	CIAT	Red
BAT477	Mesoamerican	Breeding line	CIAT	Cream
Black Hawk	Mesoamerican	Cultivar	MSU	Black
BZ16987	Mesoamerican	Breeding line	-	Cream
Carioca1070	Mesoamerican	Breeding line	CENA/USP	Carioca
DOR191	Mesoamerican	Breeding line	CIAT	Red
DOR351	Mesoamerican	Breeding line	CIAT	Purple
DOR365	Mesoamerican	Breeding line	CIAT	Carioca
DOR445	Mesoamerican	Breeding line	CIAT	Black
DOR446	Mesoamerican	Breeding line	CIAT	Black
DOR483	Mesoamerican	Breeding line	CIAT	Red
DOR500	Mesoamerican	Breeding line	CIAT	Black
DOR303	Mesoamerican	Breeding line	CIAT	Carioca (Others)
DOR364	Mesoamerican	Breeding line	CIAT	Red
EMP250	Mesoamerican	Breeding line	CIAT	Carioca
ESAL583	Mesoamerican	Breeding line	ESALQ	Carioca
FEB149	Mesoamerican	Breeding line	CIAT	Cream
FEB151	Mesoamerican	Breeding line	CIAT	Cream
FEB156	Mesoamerican	Breeding line	CIAT	Cream
FEB159	Mesoamerican	Breeding line	CIAT	Cream
G1261	Mesoamerican	Landrace	CIAT	Red
G14866	Mesoamerican	Landrace	CIAT	Black
G17666	Mesoamerican	Landrace	CIAT	Yellow
G18141	Mesoamerican	Cultivar	CIAT	Others
G2358	Mesoamerican	Landrace	CIAT	White
G2676	Mesoamerican	Cultivar	CIAT	Black
G3593	Mesoamerican	Landrace	CIAT	Red
G4002	Mesoamerican	Landrace	CIAT	Carioca (Others)
G4825	Mesoamerican	Landrace	CIAT	Carioca
G5285	Mesoamerican	Cultivar	CIAT	Red
G5902	Mesoamerican	Landrace	CIAT	Black
FEB178	Mesoamerican	Breeding line	CIAT	Carioca
G21212	Mesoamerican	Breeding line	CIAT	Black
MD732	Mesoamerican	Breeding line	IAPAR	Cream
Michigan	Mesoamerican	Cultivar	MSU	White
MUS49	Mesoamerican	Breeding line	CIAT	Red

MUS80	Mesoamerican	Breeding line	CIAT	Red
NAB87	Mesoamerican	Breeding line	CIAT	Black
NEP171	Mesoamerican	Breeding line	IICA	Black
PORRILLO70	Mesoamerican	Cultivar	CIAT	Black
PorrilloSintético	Mesoamerican	Cultivar	CIAT	Black
RIZ57	Mesoamerican	Breeding line	CIAT	Carioca
RJR21	Mesoamerican	Breeding line	-	White
RosinhaG1	Mesoamerican	-	IAC	Rosinha
RoxinhoIvaí	Mesoamerican	Landrace	-	Black
RoxodeMatoGrosso	Mesoamerican	Landrace	-	Purple
RoxodeMinas	Mesoamerican	Landrace	-	Purple
SEA5	Mesoamerican	Breeding line	CIAT	Cream
Vermelho Imbituva	Mesoamerican	Landrace	-	Red
XAN206	Mesoamerican	Breeding line	CIAT	Black
XAN236	Mesoamerican	Breeding line	CIAT	Black
LP03	Mesoamerican	Breeding line	IAPAR	Carioca
LP04	Mesoamerican	Breeding line	IAPAR	Carioca
LP05	Mesoamerican	Breeding line	IAPAR	Carioca
LP06	Mesoamerican	Breeding line	IAPAR	Carioca
LP07	Mesoamerican	Breeding line	IAPAR	Carioca
LP08	Mesoamerican	Breeding line	IAPAR	Carioca
LP09	Mesoamerican	Breeding line	IAPAR	Carioca
LP10	Mesoamerican	Breeding line	IAPAR	Carioca
LP11	Mesoamerican	Breeding line	IAPAR	Carioca
LP12	Mesoamerican	Breeding line	IAPAR	Carioca
LP13	Mesoamerican	Breeding line	IAPAR	Black
LP14	Mesoamerican	Breeding line	IAPAR	Black
LP15	Mesoamerican	Breeding line	IAPAR	Black
LP16	Mesoamerican	Breeding line	IAPAR	Black
LP17	Mesoamerican	Breeding line	IAPAR	Black
LP18	Mesoamerican	Breeding line	IAPAR	Carioca
LP19	Mesoamerican	Breeding line	IAPAR	Carioca
LP20	Mesoamerican	Breeding line	IAPAR	Black
LP21	Mesoamerican	Breeding line	IAPAR	Black
LP22	Mesoamerican	Breeding line	IAPAR	Carioca
LP23	Mesoamerican	Breeding line	IAPAR	Carioca
LP24	Mesoamerican	Breeding line	IAPAR	Carioca
LP25	Mesoamerican	Breeding line	IAPAR	Carioca
LP26	Mesoamerican	Breeding line	IAPAR	Carioca

LP27	Mesoamerican	Breeding line	IAPAR	Carioca
LP28	Mesoamerican	Breeding line	IAPAR	Carioca
LP29	Mesoamerican	Breeding line	IAPAR	Black
LP30	Mesoamerican	Breeding line	IAPAR	Black
LP31	Mesoamerican	Breeding line	IAPAR	Black
LP32	Mesoamerican	Breeding line	IAPAR	Black
LP33	Mesoamerican	Breeding line	IAPAR	Black
LP34	Mesoamerican	Breeding line	IAPAR	Carioca
LP35	Mesoamerican	Breeding line	IAPAR	Black
LP36	Mesoamerican	Breeding line	IAPAR	Carioca
LP37	Mesoamerican	Breeding line	IAPAR	Carioca
LP38	Mesoamerican	Breeding line	IAPAR	Carioca
LP39	Mesoamerican	Breeding line	IAPAR	Carioca
LP40	Mesoamerican	Breeding line	IAPAR	Black
LP41	Mesoamerican	Breeding line	IAPAR	Carioca
LP42	Mesoamerican	Breeding line	IAPAR	Carioca
LP43	Mesoamerican	Breeding line	IAPAR	Black
LP44	Mesoamerican	Breeding line	IAPAR	Black
LP45	Mesoamerican	Breeding line	IAPAR	Black
LP46	Mesoamerican	Breeding line	IAPAR	Black
LP47	Mesoamerican	Breeding line	IAPAR	Carioca
LP48	Mesoamerican	Breeding line	IAPAR	Carioca

[1]CIAT = International Center for Tropical Agriculture (Centro Internacional de Agricultura Tropical), EMBRAPA = Brazilian Agricultural Research Corporation (Empresa Brasileira de Pesquisa Agropecuária), IAC = Agronomic Institute of Campinas (Instituto Agronômico de Campinas), IAPAR = Rural Development Institute of Paraná – IAPAR – EMATER (Instituto de desenvolvimento Rural do Paraná).
